# The KDM5B and KDM1A lysine demethylases cooperate in regulating androgen receptor expression and signalling in prostate cancer

**DOI:** 10.3389/fcell.2023.1116424

**Published:** 2023-04-19

**Authors:** Veronika M. Metzler, Simone de Brot, Daisy B. Haigh, Corinne L. Woodcock, Jennifer Lothion-Roy, Anna E. Harris, Emeli M. Nilsson, Atara Ntekim, Jenny L. Persson, Brian D. Robinson, Francesca Khani, Kristian B. Laursen, Lorraine J. Gudas, Michael S. Toss, Srinivasan Madhusudan, Emad Rakha, David M. Heery, Catrin S. Rutland, Nigel P. Mongan, Jennie N. Jeyapalan

**Affiliations:** ^1^ Biodiscovery Institute, University of Nottingham, Nottingham, United Kingdom; ^2^ COMPATH, Institute of Animal Pathology, University of Bern, Bern, Switzerland; ^3^ Department of Oncology, University Hospital Ibadan, Ibadan, Nigeria; ^4^ Department of Molecular Biology, Umeå University, Umeå, Sweden; ^5^ Department of Biomedical Sciences, Malmö Universitet, Malmö, Sweden; ^6^ Department of Urology, Weill Cornell Medicine, New York, NY, United States; ^7^ Department of Pharmacology, Weill Cornell Medicine, New York, NY, United States; ^8^ School of Pharmacy, University of Nottingham, Nottingham, United Kingdom

**Keywords:** epigenetics, splicing, transcriptional regulation, KDM-inhibitors, histone modification

## Abstract

Histone H3 lysine 4 (H3K4) methylation is key epigenetic mark associated with active transcription and is a substrate for the KDM1A/LSD1 and KDM5B/JARID1B lysine demethylases. Increased expression of KDM1A and KDM5B is implicated in many cancer types, including prostate cancer (PCa). Both KDM1A and KDM5B interact with AR and promote androgen regulated gene expression. For this reason, there is great interested in the development of new therapies targeting KDM1A and KDM5B, particularly in the context of castrate resistant PCa (CRPC), where conventional androgen deprivation therapies and androgen receptor signalling inhibitors are no longer effective. As there is no curative therapy for CRPC, new approaches are urgently required to suppress androgen signalling that prevent, delay or reverse progression to the castrate resistant state. While the contribution of KDM1A to PCa is well established, the exact contribution of KDM5B to PCa is less well understood. However, there is evidence that KDM5B is implicated in numerous pro-oncogenic mechanisms in many different types of cancer, including the hypoxic response, immune evasion and PI3/AKT signalling. Here we elucidate the individual and cooperative functions of KDM1A and KDM5B in PCa. We show that KDM5B mRNA and protein expression is elevated in localised and advanced PCa. We show that the KDM5 inhibitor, CPI-455, impairs androgen regulated transcription and alternative splicing. Consistent with the established role of KDM1A and KDM5B as AR coregulators, we found that individual pharmacologic inhibition of KDM1A and KDM5 by namoline and CPI-455 respectively, impairs androgen regulated transcription. Notably, combined inhibition of KDM1A and KDM5 downregulates AR expression in CRPC cells. Furthermore, combined KDM1A and KDM5 inhibition impairs PCa cell proliferation and invasion more than individual inhibition of KDM1A and KDM5B. Collectively our study has identified individual and cooperative mechanisms involving KDM1A and KDM5 in androgen signalling in PCa. Our findings support the further development of KDM1A and KDM5B inhibitors to treat advanced PCa. Further work is now required to confirm the therapeutic feasibility of combined inhibition of KDM1A and KDM5B as a novel therapeutic strategy for targeting AR positive CRPC.

## 1 Introduction

The androgen receptor (AR, NR3C4) is the key driver of prostate cancer (PCa) initiation and progression (Hoang et al., 2017; Takeda et al., 2018; Duan et al., 2019). For this reason, androgen deprivation therapies (ADT) and AR signaling inhibitors (ARSI) remain the important treatments for advanced PCa (Hoang et al., 2017). ADT and ARSI inhibit androgen driven PCa (de Brot et al., 2015), but the emergence of castrate-resistant PCa (CRPC) where the AR continues to orchestrate pro-oncogenic signaling occurs frequently in ADT-treated patients (Sharma et al., 2013). The emergence of castrate-resistance and disease progression, and indeed the increasing numbers of treatment-emergent neuroendocrine PCa (NePC) which lack AR signaling (Mucci et al., 2000; Humphrey, 2012; Dang et al., 2015), represents major therapeutic challenges (Beltran et al., 2014). Recently, clinical studies showed improved outcomes for the combination of ADT with next-generation AR antagonists in both non-metastatic and metastatic CRPC (Cattrini et al., 2019; Mori et al., 2020; Sathianathen et al., 2020), but therapy resistance still emerges. For these reasons there is an urgent clinical need to develop novel therapeutic approaches that both block androgen signaling in PCa cells and prevent or bypass the emergence of castrate resistance (Siegel et al., 2020).

The transcriptional functions of the AR depend on the recruitment of multiple, enzymatically distinct, epigenetic coregulators, including lysine demethylases (KDMs) which cooperate to regulate transcription of AR-regulated genes (Metzger et al., 2005; Kahl et al., 2006; Wissmann et al., 2007). Furthermore, the expression of AR, in turn, is subject to epigenetic regulation by lysine demethylase KDM1A (Cai et al., 2011; Cai et al., 2014). We and others identified an additional level of reciprocal regulation whereby hsa-mIR-137 is upregulated by androgen in non-malignant prostate cells and functions to limit expression of notable AR coregulators, including the lysine demethylase coregulators KDM1A, JMJD2A/KDM2A and NCoA2/SRC-2 (Shin and Janknecht, 2007; Kauffman et al., 2011; Althoff et al., 2013; Nilsson et al., 2015). However, expression of hsa-miR-137 is epigenetically silenced in PCa, thereby enabling increased expression of AR coregulators and by inference, resulting in amplification of transcription mediated by the AR-coregulator complex (Nilsson et al., 2015). Collectively these data indicate complex regulation of the expression and function of the AR-coregulator complex in non-malignant prostate and PCa cells.

The importance of lysine demethylases in AR signaling and the availability of pharmacological inhibitors renders the KDMs therapeutic targets in PCa, including in CRPC. Crucial roles for KDM1A in PCa are well established (Kashyap et al., 2013; Nilsson et al., 2015). KDM1A can function to demethylate both activating and repressive H3 marks, specifically mono- and di-methylated histone H3 lysine 4 (H3K4) and histone H3 lysine 9 (H3K9) and is important in AR transcriptional regulation (Metzger et al., 2005; Metzger et al., 2010; Cai et al., 2014). The functional and clinical relevance of KDM5B in PCa is less well understood. KDM5B (JARID1B/PLU1), is also regulated by hsa-miR137 (Nilsson et al., 2015) and functions to demethylate H3K4 (Xiang et al., 2007; Klein et al., 2014). Expression of *KDM5B* is increased in several cancer types (Dai et al., 2014; Wang et al., 2015; Bamodu et al., 2016), including PCa (Xiang et al., 2007), with elevated expression implicated in multiple pro-oncogenic mechanisms in many cancer types (Hayami et al., 2010; Krishnakumar and Kraus, 2010; Dai et al., 2014; Shen et al., 2015; Zhao and Liu, 2015; Wang et al., 2016a; Bamodu et al., 2016; Wang et al., 2016b). Like KDM1A (Metzger et al., 2005; Cai et al., 2011), KDM5B has been reported to both interact with AR, promote AR signaling (Xiang et al., 2007) and regulate AR promoter activity in certain contexts (Casati et al., 2013). More recently, KDM5B was shown to be required for hyperactivation of the PI3K/AKT signaling (Li et al., 2020), supporting therapeutic targeting KDM5B in PCa. In contrast, a prostate-specific conditional KDM5B knock-out mouse suggested contrasting roles for KDM5B in prostate development and carcinogenesis (Liu et al., 2020). Despite the mechanistic relevance of KDM5B as a novel therapeutic target in PCa, understanding the functional and clinical relevance of KDM5B, and how the KDM1A and KDM5B H3K4 demethylases may cooperate in PCa remains incomplete. For this reason, we have completed a clinical and mechanistic analysis of KDM5B in PCa cell lines, prostate adenocarcinoma and neuroendocrine tumor patient specimens and examined the effect of individual and cooperative inhibition of KDM1A (Willmann et al., 2012) and KDM5B (Vinogradova et al., 2016) on androgen regulated gene expression. We also examine the effect of KDM5 family inhibition on cellular proliferation and invasion of PCa cells. Our results reveal that cooperate inhibition of KDM1A and KDM5 was required for downregulation of AR in CRPC.

## 2 Materials and methods

### 2.1 Ethics statement and tissue specimens

The project was conducted under the oversight of the local ethics committee of the University of Nottingham School of Veterinary Medicine and Science (approvals#: 3483 211102; 1533 150901; 1861 161006) and the Weill Cornell Medicine Institutional Review Board (approval: 1008011210). For the Nottingham cohort (Cohort 1), a series of prostatectomy specimens from prior to 2006 were obtained from the Nottingham University Hospital biobank (approval#:ACP0000184) and a tissue microarray comprising non-malignant prostate (*n* = 43) and PCa (*n* = 97) specimens prepared using standard methods. Tumor Gleason grades were reported by specialist urologic pathologists at the time of diagnosis. Demographics and clinical characteristics of study participants are provided (Table 1). The Weill Cornell cohort (Cohort 2) contained post-mortem specimens of metastatic (*n* = 40) and neuroendocrine PCa (NEPC; *n* = 13). Metastatic tumors were located at several locations over the body, including brain, bone and liver. NEPC tumors were in the prostate. No further clinical parameters were available. The Helsinki Declaration of Human Rights and the UK Human Tissue Act were strictly observed.

**TABLE 1:** Cohort 1—Nottingham TMA clinical parameters.

Clinicopathological parameters	Frequency N (%)
**Age**	
<60	44 (42.3%)
≥60	60 (57.7%)
**Race**	
White	94 (95.9%)
Mixed/Black Carribean	2 (2.05%)
Other	2 (2.05%)
**Gleason**	
6	12 (11.7%)
≥7	91 (88.3%)
**pTNM**	
T1 and T2	66 (66.7%)
T3	33 (33.3%)
**BCR**	
No	59 (64.1%)
Yes	33 (35.9%)

### 2.2 Bioinformatic analysis of KDM5B in clinical specimens

The cBioPortal for Cancer Genomics was used to study genetic alterations in KDM5B in primary and metastatic prostate adenocarcinoma, and neuroendocrine carcinoma patient specimens (https://www.cbioportal.org/) (Cerami et al., 2012). Overall survival and the disease and progression-free survival regarding alterations were calculated by Kaplan-Meier estimate using the cBioPortal. Three studies were included in the analysis: i) TCGA Prostate Adenocarcinoma study (n = 499) (Cancer Genome Atlas Research et al., 2015), ii) the SU2C/PCF Dream Team Metastatic Prostate Adenocarcinoma study (*n* = 444) (Abida et al., 2019) and iii) the Trento/Cornell/Broad Neuroendocrine PCa study (*n* = 114) (Beltran et al., 2016). PRAD TCGA transcript data was utilized. Patients were categorized into quartiles based on normalized KDM5B expression and differential analysis was performed using DESeq2 (Love et al., 2014), comparing global gene expression in patients with low (quartile 1) *versus* high (quartile 4) KDM5B expression. Pathway analysis was carried out using WebGestalt 2017 (Wang et al., 2017).

### 2.3 Immunohistochemistry

The expression and clinical-pathologic associations of KDM5B expression were assessed using immunohistochemistry as described (Kashyap et al., 2013). Briefly, 4 μm sections of the Nottingham and Weill Cornell PCa TMAs were incubated with the anti-KDM5B antibody (1:50 dilution; #H00010765-M02, Abnova, Taipei City, Taiwan) and signal detected using the Novolink Max Polymer Detection system (Leica Biosystems; Milton Keynes, United Kingdom). High resolution slide scans were prepared for each stained TMA and staining assessed using a modified Histo-score (H-score) considering both intensity and % cell positivity on a range of 0–300. Both nuclear and cytoplasmic staining were analysed, and a proportion of cores (10%) independently assessed to identify inter-observer variability. Discordant scores reviewed by both observers in consultation with an expert histopathologist (MST). For H scores, *p*-values were determined by χ2-test (asymptotic significance, 2-sided) using IBM^®^ SPSS^®^ Statistics, Version 24. Statistical significances of Kaplan-Meier estimates were calculated using the log-rank (Mantel-Cox) test. Scoring reliability between two independent scorers was analysed by both Cronbach’s alpha in SPSS and Spearman’s rank-order correlation.

### 2.4 Cell lines and culture conditions

The immortalized non-malignant human prostate epithelial cell line PNT1A was provided by Dr. Jenny Persson (Umeå University). LNCaP were purchased from the European Collection of Authenticated Cell Cultures via United Kingdom Health Security Agency (formerly Public Health England), 22Rv1 (#CRL-2505), PC3 (#CRL-1435) and Du145 (#HTB-81) were obtained from ATCC and LNCaP-C4-2 (#CRL-3314) were a generous gift from Dr. Doug Scherr, Department of Urology, Weill Cornell Medicine. All cell lines were maintained in 5% CO2 in phenol red containing RPMI-1640 medium supplemented with 10% fetal bovine serum (FBS, 1% penicillin-streptomycin, 2 mM L-Glutamine and 1 mM sodium pyruvate. For androgen (R1881) experiments, cells were grown in phenol red-free RPMI-1640 medium supplemented with 1% penicillin-streptomycin, 2 mM L-Glutamine and 1 mM sodium pyruvate and either 10% hormone depleted dialyzed or charcoal-stripped FBS. For androgen treatments, cells were treated for 72 h with 1 nM R1881 (Sigma Aldrich) dissolved in ethanol, and 0.1% ethanol treatments were used as vehicle control. KDM5B selective inhibitor CPI-455 Cayman Chemical (Michigan, United States) (Vinogradova et al., 2016) and KDM1A inhibitor Namoline (Abcam, Cambridge, United Kingdom) were dissolved in 100% DMSO and used at concentrations 25–50 µM. Final DMSO concentrations for all experiments were <0.1%.

### 2.5 SiRNA mediated functional depletion and pharmaco-inhibition of KDM5B.

Functional depletion of KDM5B in PCa cells was performed using ON-TARGETplus siRNA SMART pools (DharmaconTM, Lafayette, CO) using for human KDM5B (#L-009899-00–0005). ON-TARGETplus non-targeting “scramble” control siRNAs (#D-001810-10-05, GE DharmaconTM) were employed as negative controls. Cells were transfected using DharmaFECT-2 Transfection Reagent (GE DharmaconTM) and transfection was performed after manufacturer’s instructions. The effect of KDM5B inhibitors on PCa cell proliferation was measured by treating PCa cells with CPI-455, alone or in combination with R1881 and cell numbers were measured after 3 and 6 days using a CyQUANT NF Cell Proliferation Assay Kit (Invitrogen) and fluorescence measurement was performed with excitation at ∼485 nm and emission detection at ∼530 nm in the Varioskan Flash plate reader (Thermo Fisher Scientific, Massachusetts, United States).

### 2.6 Gene expression analysis

For gene expression analysis, PCa cells were plated in 6-well plates (Greiner Bio-one) and grown until 60%–70% confluent before treatment. Cells were treated with CPI-455 (25 μM, 50 µM) and/or R1881. Cells were treated CPI-455 and Namoline then cells harvested for RNA extraction using Trizol (Ambion) or RNeasy kits (Qiagen) and cDNA synthesised using Quanta cDNA synthesis kits (VWR). For mRNA expression analysis, hydrolysis probe based real-time quantitative polymerase chain reaction (RT-qPCR) was performed with the following TaqmanTM probes (ThermoScientific, Massachusetts, United States): KDM5B Hs00981910_m1; GAPDH Hs03929097_g1; PSA/KLK3 Hs02576345_m1; TMPRSS2 Hs01122322_m1; VEGFA Hs00900055_m1; AR Hs00171172_m1; FOXA1 Hs04187555_m1; NKX3.1 Hs00171834_m1. GAPDH was used as a house keeping gene. The qRT-PCR reactions were performed in a LightCycler 480 II (Roche) instrument as described (Nilsson et al., 2015). For multiple comparisons one-way ANOVA was used by comparing the mean of each column with the mean of every other column with no matching or pairing and corrected for multiple testing using the Bonferroni *post hoc* method. For comparison of two means, parametric t-tests were carried out. Non-parametric t-test (Welch’s) was utilized for comparisons between cell-lines and for drug-treatments within the same cell-line t-test was performed. In general, ≥2-fold difference in mRNA considered biologically significant and *p*-values ˂0.05 were considered statistically significant with a confidence interval of 95%.

### 2.7 Immunoblotting

For western analysis, cells were washed with 1X phosphate-buffered saline (PBS) and harvested in final sample buffer (100 mM Tris-HCl pH 6.8, 4% SDS, 20% glycerol) and stored at −80°C. Protein concentration was measured using the DCTM (detergent compatible) Protein Assay (BIO-RAD) and were diluted to 800–1500 μg/μL stock solutions for Western blot analysis and stored at −80°C. Protein samples (10–20 µg) were diluted with 5X Laemmli loading buffer and boiled at 95°C for 5 min before loading and PAGE after which proteins were transferred from the gel onto a polyvinylidene difluoride (PVDF) membrane (Immobilon-P Membrane, 0.45 µm, Merck) via semi-dry blotting. The membrane was blocked using either 5% BSA or 3% milk as indicated for the respective antibodies. The antibodies and dilutions used were rabbit anti-hKDM5B (Cell Signaling # 3273, 1/1000); mouse monoclonal anti-GAPDH (Abcam, ab9484, 1/5000) and mouse monoclonal anti-actin (Invitrogen, MA515739, 1/10,000). Goat Anti-Mouse IgG (ab97023, Abcam, Cambridge, United Kingdom) or Goat Anti-Rabbit IgG (ab6721, Abcam) were used as secondary antibodies and signal detected using Amersham TM ECL Prime reagent (GE Healthcare, Chicago, United States) and image captured using a ChemiDoc TM MP Imaging System (BIO-RAD, Caifornia, United States) and the signal intensity quantified using Image studio Lite software (Licor).

### 2.8 RNA seq analysis

RNA Seq analysis was performed in biological replicates of PCa cells treated with vehicle (DMSO, 0.05%) plusR 1881 (1 nM) and R1881 (1 nM) and CPI-455 (50 µM). Fastq files were quality processed (phred score >30 retained) and adapters trimmed using TrimGalore. The QC-processed reads were aligned to the human Ensembl annotated reference genome (GRCh38) using the STAR aligner. Differential gene expression were calculated using FeatureCounts (Liao et al., 2014) and EdgeR (Robinson et al., 2010) as described in Minton et al., 2016 (Minton et al., 2016). Pathway analysis was performed using WebGestalt (Wang et al., 2017). For pathway analysis the RNASeq data was filtered according to log2FC ≥ 1 and ≥−1 respectively. Differential splicing analysis was conducted using rMATs (Shen et al., 2014). RNA-Seq data is available from NCBI-GEO under the following accession: GSE194278.

## 3 Results

### 3.1 KDM5B alterations increase with PCa progression

**FIGURE 1:**
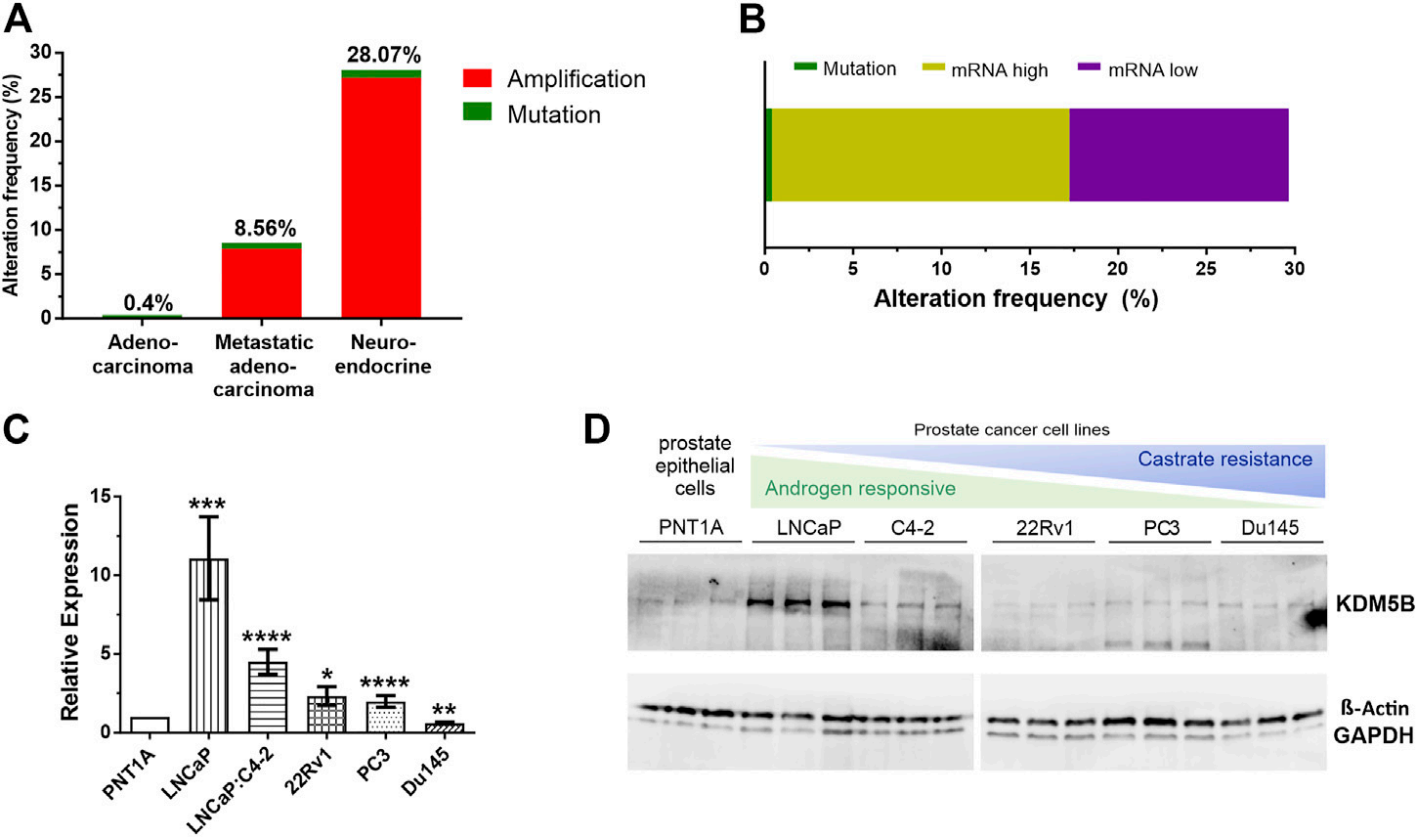
KDM5B alterations in prostate cancer (PCa) patients. **(A)** Percentage of *KDM5B* alterations in a comparative analysis of prostate cancer patients from the TCGA Provisional Prostate Adenocarcinomas, the SU2C/PCF Dream Team Metastatic Prostate Adenocarcinomas and the Trento/Cornell/Broad Neuroendocrine Prostate Cancer studies (total *n* = 1057). **(B)** Percentage mutations and *KDM5B* mRNA levels in the 147 TCGA PCa patients with *KDM5B* alterations (RNA Seq V2 RSEM, mRNA expression *z*–score = 1). **(C)** *KDM5B* mRNA levels in PCa cell lines compared to non-malignant epithelial cells, PNT1A. Significant higher expression shown for LNCaP, LNCaP: C4-2, 22RV1, and AR negative PC3. DU145 expression was significantly lower than PNT1A. **(D)** Immunoblot showing KDM5B protein levels in PCa cell lines, both Beta-Actin and GAPDH used as loading controls. Significance shown as *p ≤ 0.05, **p ≤ 0.005, ***p ≤ 0.001, *p ≤ 0.0001.

Using cBioPortal (Cerami et al., 2012), we analysed *KDM5B* gene alterations in the TCGA PRAD prostate adenocarcinoma study (*n* = 499), the SU2C/PCF Dream Team Metastatic Prostate Adenocarcinoma study (n = 444) and the Trento/Cornell/Broad Neuroendocrine PCa study (*n* = 114). Comparison of the 1057 tumors showed *KDM5B* was most frequently altered in neuroendocrine tumors (28.07%), followed by metastatic prostate adenocarcinoma (8.56%) and TCGA PRAD cohort (0.4%), with the majority of alterations consisting of gene amplification (Figure 1A). In the TCGA PRAD cohort analysis of mRNA expression of *KDM5B* was altered in 29.2% tumors (147/499 cases) of which 16.8% had high mRNA levels and 12.4% had low mRNA levels (Figure 1B). None of the cases had copy number alterations and only two cases possessed a *KDM5B* coding variant, suggesting *KDM5B* changes at the transcriptional level in adenocarcinomas are not attributable to sequence or copy number alterations. This was confirmed by GISTIC analysis correlation with mRNA levels in the TCGA PRAD cohort, where *KDM5B* levels varied greatly within tumors with diploid copy number and a significant weak correlation (*n* = 147; Pearson correlation 0.29 *p* = 3.14e-11); (Supplementary Figure S1A). Utilizing the PRAD TCGA data, a comparison of global gene expression associated in patients with low *KDM5B* (quartile 1, Q1) as compared to patients with high *KDM5B* (quartile 4, Q4) was completed. Expression of 7088 transcripts (5458 genes) were higher in patients with low *KDM5B*, and expression of 9024 transcripts (5463 genes) were lower in patients with low *KDM5B* as comparted to patients with high KDM5B (Supplementary Table S1A). Pathway analysis identified genes expressed higher in tumors with high *KDM5B* (Q4) to be involved in cancer pathways, including PCa. Genes that were expressed higher in low *KDM5B* tumors (Q1) were involved in metabolic signaling pathways (Supplementary Table S1A).

KDM5B levels were analysed in PCa and CRPC cell lines. *KDM5B* expression was generally higher in PCa cell lines when compared to prostate epithelial PNT1A cells (Figure 1C). At the protein level, KDM5B was expressed in all PCa cell lines, but interestingly was higher in AR-dependent LNCaP cells as compared to prostate epithelial cells, PNT1A (Figure 1D). Treatment of PCa cell lines with synthetic androgen, R1881, for 72 h did not affect *KDM5B* expression in the androgen-responsive LNCaP and LNCaP:C4-2 cells (Supplementary Figure S1B) indicating *KDM5B* is not androgen regulated.

### 3.2 KDM5B in PCa specimens and clinical relevance

**FIGURE 2:**
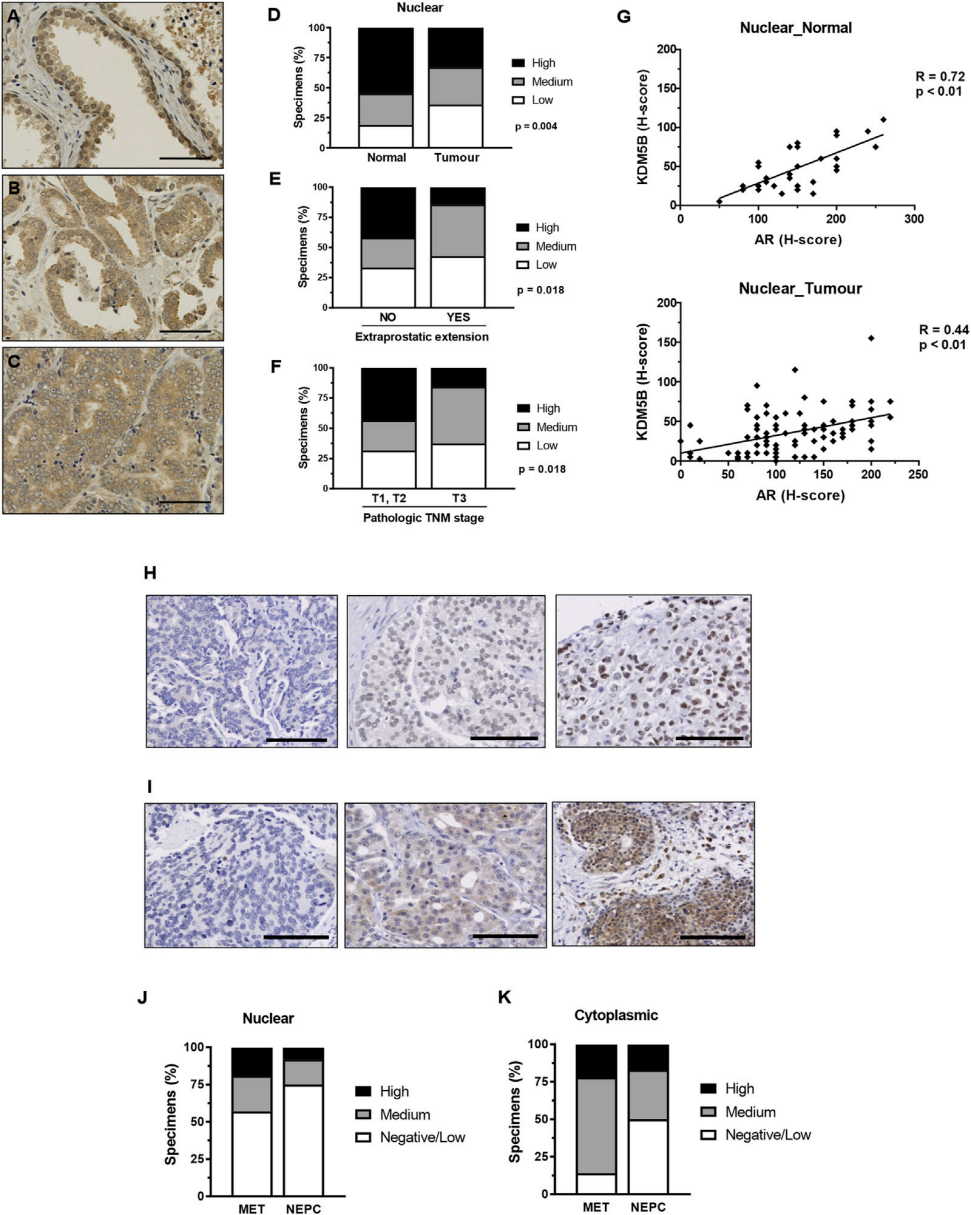
KDM5B in PCa specimens. Immunohistochemistry analysis of KDM5B protein in non-malignant, benign, primary and metastatic adenocarcinomas plus neuroendocrine prostate tumors. Cohort 1_non-malignant/normal (*n* = 43) and prostate adenocarcinomas (*n* = 97) specimens. Representative KDM5B staining of non-malignant tissue **(A)**, adenocarcinoma **(B)** and less differentiated tumour tissue **(C)** nuclear staining, scale bar = 50 µM. **(D)** Nuclear H-scores were split into 3 groups, low (0–20), medium (25–40) and high (45–155), % number of specimens/group are shown. SPSS analysis was performed using clinical data to correlate clinical parameters with KDM5B intensities in the tumours (*n* = 97). Significant differences in the level of KDM5B was identified with **(E)** extraprostatic extension and **(F)** TMN staging. Statistical *p*-values were determined by χ2-test. TNM = tumour, node, metastasis, scale bar = 100 µM. **(G)** KDM5B nuclear H-score correlation with AR nuclear H-score in non-malignant normal tissue (*n* = 33) and tumour specimens (*n* = 95). Pearson’s correlation with *p*-values is shown. Cohort 2 consisted metastatic (*n* = 40) and NEPC (*n* = 13). Representation of low to high (Left to right) levels of nuclear **(H)** and cytoplasmic **(I)** staining is shown. Nuclear **(J)** and cytoplasmic **(K)** H-scores were split into 3 groups, Negative/low (<50), medium (50–100) and high (>100).

We investigated the clinical relevance of KDM5B. We utilized immunohistochemistry to examine KDM5B protein expression in two PCa cohorts that contained benign prostate tissue, primary adenocarcinoma, metastatic and neuroendocrine prostate specimens. Nuclear and cytoplasmic KDM5B expression was observed and quantified using the H-score method. Cohort 1 contained a TMA of non-malignant tissue and adenocarcinomas (Table 1), of which 43 non-malignant and 97 adenocarcinoma specimens were scored and taken forward for analysis. KDM5B expression was observed as expected in the nucleus, but also in the cytoplasm of non-malignant and tumor specimens (Figures 2A–C; Supplementary Figures S2A–C). KDM5B staining was evaluated using H-scores. Scores were divided into 3 equal groups, for nuclear (low 0–20, medium 25–40, high 45–155) and cytoplasmic staining (low 25-50, medium 55-75, high 80-150). In comparison to non-malignant tissue, nuclear KDM5B staining in prostate tumor was significantly lower (*p* < 0.05; Figure 2D), with no significant difference seen in cytoplasmic staining (Supplementary Figures S2D). Kaplan-Meier estimates were performed to determine whether KDM5B expression in human PCa tissue correlated with biochemical recurrence (BCR). There were no significant differences with BCR between low *versus* high KDM5B expression in the nucleus or cytoplasm (Supplementary Figures S2E, F). We additionally correlated KDM5B nuclear and cytoplasmic H-scores with clinical parameters. No statistical significance was identified with age, Gleason score, high grade prostatic intraepithelial neoplasia (PIN), perineural invasion or pre-operative PSA (data not shown) in relation to nuclear and cytoplasmic H-scores. Interestingly, high KDM5B expression in the nucleus significantly correlated with absence of extraprostatic extension and low TNM stage (T1, T2; no extraprostatic extension, *p* < 0.05; Figures 2E, F). These findings suggest that KDM5B is present in a subset of tumors, but loss of KDM5B is associated with PCa progression.

As KDM5B acts as an AR coregulator we next correlated AR expression with KDM5B in PCa specimens in cohort 1. Expression of KDM5B showed positive correlation with AR in non-malignant (R = 0.72, *p* < 0.01) and tumor specimens (R = 0.44, *p* < 0.01, Figure 2G). We then analyzed KDM5B in cohort 2 which contained metastatic and neuroendocrine specimens (*n* = 53). Nuclear and cytoplasmic KDM5B was identified in a subset of advanced PCa (Figures 2H, I). H-scores were dichotomized into low <50, medium 50–100, high >100 for both nuclear and cytoplasmic expression. There was a subset of tumors that contained high nuclear KDM5B expression (19% metastatic and 8% Neuroendocrine, Figures 2J, K). Interestingly, KDM5B was detected in the cytoplasm at higher levels compared to nuclear staining in metastatic tumors (Figure 2K). A positive correlation between nuclear and cytoplasmic staining (R = 0.398, N = 40, *p* = 0.01) was seen, this suggests that in some cases KDM5B is being retained in the cytoplasm. Overall, these findings suggest that KDM5B is crucial for initial AR–dependent tumor growth and could also play a role in metastatic tumors once established.

### 3.3 Functional inhibition of KDM5B demethylase affects expression of AR regulated genes

**FIGURE 3:**
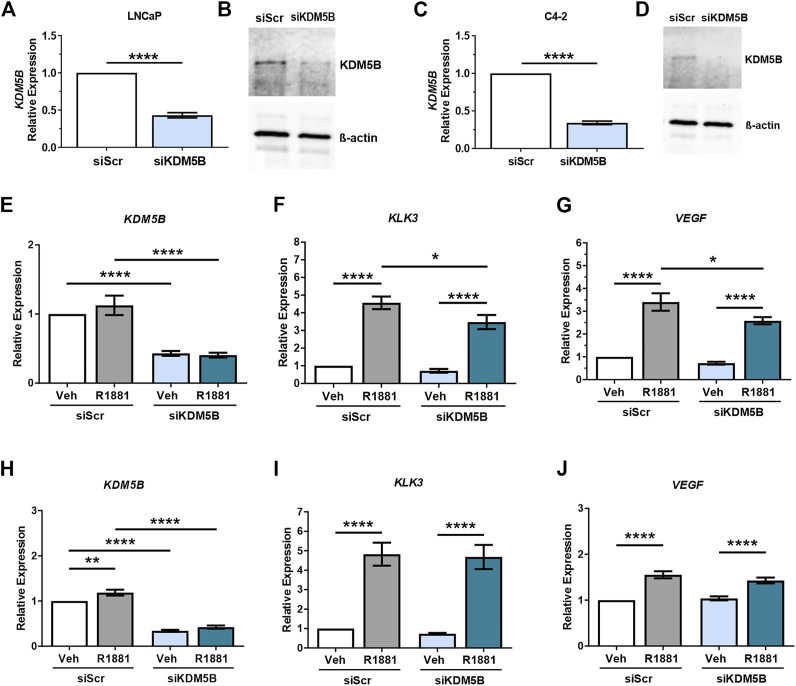
SiRNA-mediated knockdown of KDM5B in PCa cell lines LNCaP and C4-2. Confirmation of siRNA knockdown of *KDM5B* mRNA levels by RT-qPCR **(A,C)** and immunoblot for KDM5B protein levels. Beta-Actin was used as loading control **(B,D)**. Relative expression analysis on the effect of androgen treatment (1 nM R1881) and KDM5B knockdown on AR target genes, KLK3, and VEGF for LNCaP **(E–G)** and LNCaP:C4-2 **(H–J)**. Relative expression was calculated against vehicle scrambled control and normalization performed using GAPDH. Veh–Vehicle, R1881–1 nM R1881, siScr–siRNA scrambled control, siKDM5B- *KDM5B* siRNA smart pool. Significance *p* values shown as *p ≤ 0.05, **p ≤ 0.005, ***p ≤ 0.001, *p ≤ 0.0001.

To elucidate the role of KDM5B in PCa, we used siRNAs to functionally deplete KDM5B in castrate sensitive LNCaP and castrate resistant LNCaP:C4-2 cells. Knockdown was performed for 72 h and confirmed at the mRNA and protein levels, with significant reduction (60%–70%) in both *KDM5B* mRNA (Figures 3A, C) and KDM5B protein levels (Figures 3B, D). Given the putative role for KDM5B as an AR coregulator (Xiang et al., 2007), we assessed the effects of KDM5B knockdown on androgen (R1881) regulated genes. Expression of *KDM5B* was unchanged by androgen (Supplementary Figure S1A) and did not affect the levels of *KDM5B* knockdown (Figures 3E, H). KDM5B knockdown impaired androgen induction of KLK3/PSA and VEGF in parental androgen-dependent LNCaP (Figures 3F, G), but not in the LNCaP: C4-2 CRPC derivative (Figures 3I, J). This suggests that KDM5B can act to promote AR signaling at a subset of AR-regulated genes and in certain cellular contexts.

**FIGURE 4:**
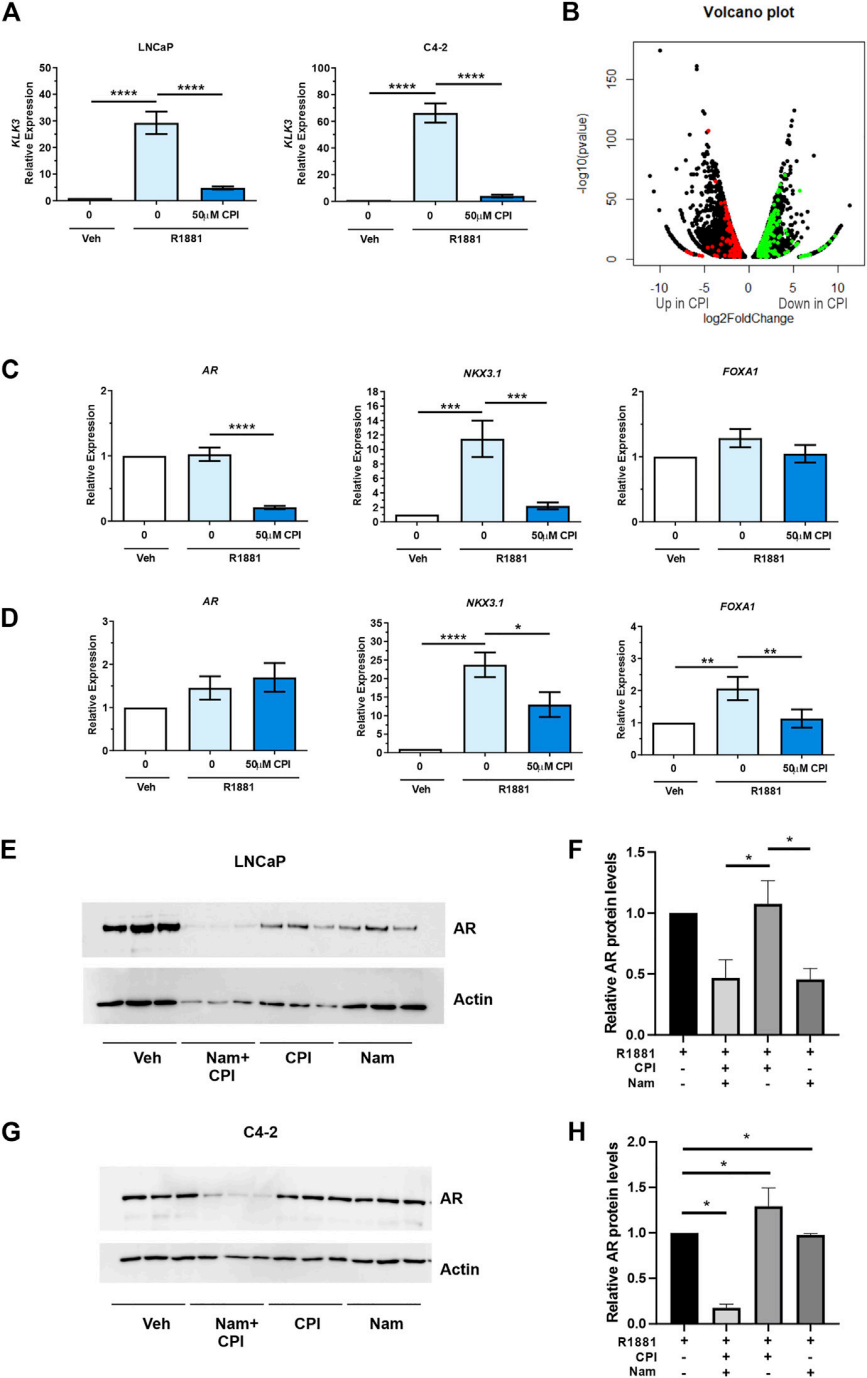
KDM5 inhibitor treatment effects on AR and the transcriptome in PCa cell lines LNCaP and C4-2. CPI-455 (CPI) treatment in the presence of synthetic androgen R1881 (1 nM) was performed for 72 h. **(A)** RT-qPCR of AR target gene *KLK3* levels (normalised to *GAPDH*). Androgen induction significantly attenuated with CPI-455 treatment. **(B)** Volcano plot of the differentially expressed genes between vehicle + R1881 and CPI-455 + R1881treated LNCaP cells. A negative log2 fold change (FC) shows genes upregulated with CPI-455 treatment and positive log2 FC value genes downregulated with CPI-455 treatment. Red and green spots highlight AR target genes that are up or downregulated by CPI treatment respectively. **(C,D)** RT-qPCR validation of *AR* and AR target gene, *NKX3.1* and pioneer factor *FOXA1* in both LNCaP **(C)** and LNCaP: C4-2 **(D)**. **(E,G)** Immunoblot showing the AR levels after 72 h of KDM inhibitor treatments for LNCaP and LNCaP:C4-2 respectively. Relative AR protein levels for LNCaP **(F)** and LNCaP: C4-2**(H)** were calculated by normalising to Actin signal and calibrating to vehicle (*n* = 3, for each treatment). Veh–Vehicle control 0.1% DMSO, CPI–CPI- 455 (50 µM), Nam–Namoline (50 µM). Significance shown as *p*-values *p ≤ 0.05, **p ≤ 0.005, ***p ≤ 0.001, *p ≤ 0.0001.

We next evaluated the effect of CPI-455, a KDM5 family inhibitor in PCa (Vinogradova et al., 2016). CPI-455 was shown to preferentially inhibit KDM5B (IC50 0.003), then KDM5A (IC50 0.01), and KDM5C (IC50 0.03), with concentrations >6.5 µM inducing an increase in H3K4me3 (Vinogradova et al., 2016). KDM5A and KDM5C have been shown to be expressed higher in PCa than non-malignant tissues. While both KDM5A and KDM5C are expressed in LNCaP cells, their expression is higher in AR negative cell lines, PC3 and DU145 (Stein et al., 2014; Hong et al., 2019; Du et al., 2020). KDM5A increased cell proliferation and invasion (Du et al., 2020) and KDM5C promotes EMT in PCa cells (Lemster et al., 2022), but neither have been shown to act as an AR co-regulator. We firstly confirmed CPI-455 (50 µM) for 72 h attenuated androgen induced expression of the prototypical AR target gene, *KLK3/PSA* (Figure 4A). We next used RNA sequencing to determine the transcriptome wide effects of CPI-455 on androgen (R1881) induced gene expression. In total 11735 genes were identified as significantly differentially expressed (≥2-fold change, FDR *p* < 0.05), of which 6,626 genes were upregulated and 5416 genes downregulated with CPI-455 treatment (Figure 4B; Supplementary Table S2A). To investigate changes to the AR-regulated transcriptome, AR-regulated genes identified by Sharma and colleagues (Sharma et al., 2013) were examined against differentially expressed genes upon CPI-455 treatment. Of these 1371 AR-regulated genes, expression of 486 genes were significantly affected by KDM5B inhibition in LNCaP cells (≥2-fold, FDR q < 0.05), with expression of 351 (72%) genes downregulated with CPI-455 treatment (Supplementary Table S2B). Crucially, mRNA expression of AR was significantly (≥2FC, q < 0.05) reduced by CPI-455 (Supplementary Figure S2B). To confirm this, we used RT-qPCR to examine expression of *AR*, known *AR* target *NKX3.1* and pioneer factor involved in AR target gene expression, *FOXA1*. In LNCaP, the expression of *AR* and *NKX3.1* but not *FOXA*1 were reduced by CPI-455 (Figure 4C). In contrast in LNCaP:C42, *KDM5B* inhibition impaired androgen induction of *NKX3.1* and also downregulated *FOXA1*, whereas *AR* expression was unchanged (Figure 4D).

Over representation analysis of KEGG pathways was undertaken on the differentially expressed genes (11735) to identify pathways that were altered following CPI-455 treatment (Table 2). Genes upregulated with CPI-455 treatment are involved with the ribosome and in neurological disorders (FDR *p* < 0.01). More importantly, CPI-455 downregulated genes involved in several key cancer-related pathways including Hippo, WNT and PI3K/AKT pathways (FDR *p* < 0.01, Table 2). These results align with the PRAD TCGA tumor analysis showing that high *KDM5B* expression is associated with cancer-promoting pathways (Supplementary Table S1B). Overall, these findings show that pharmacological inhibition of KDM5 family, downregulated the tumor-promoting transcriptome in AR-dependent PCa cells.

**TABLE 2:** Pathway analysis of differentially expressed genes (fold change log2 ≥ 1 and FDR - *p*-value ≤0.05) with CPI-455 treatment in LNCaP.

Kegg pathways		No. Of genes	FDR *p*-value
*Upregulated with CPI treatment (FDR < 0.05)*			
hsa03010	Ribosome—*Homo sapiens* (human)	53	4.80E-13
hsa00190	Oxidative phosphorylation—*Homo sapiens* (human)	40	7.02E-06
hsa05016	Huntington’s disease—*Homo sapiens* (human)	49	5.33E-05
hsa05012	Parkinson’s disease—*Homo sapiens* (human)	36	1.35E-03
hsa05322	Systemic lupus erythematosus—*Homo sapiens* (human)	33	5.23E-03
hsa05010	Alzheimer’s disease—*Homo sapiens* (human)	39	5.28E-03
hsa04932	Non-alcoholic fatty liver disease (NAFLD)—*Homo sapiens* (human)	33	3.40E-02
*Downregulated with CPI treatment (FDR < 0.01)*			
hsa05200	Pathways in cancer	149	3.05E-10
hsa04390	Hippo signaling pathway	66	1.08E-06
hsa05224	Breast cancer	58	1.31E-04
hsa04144	Endocytosis	89	3.70E-04
hsa04141	Protein processing in endoplasmic reticulum	62	3.85E-04
hsa05222	Small cell lung cancer	37	6.71E-04
hsa05206	MicroRNAs in cancer	56	7.79E-04
hsa00510	N-Glycan biosynthesis	24	1.31E-03
hsa05205	Proteoglycans in cancer	69	1.96E-03
hsa04550	Signaling pathways regulating pluripotency of stem cells	52	1.96E-03
hsa04512	ECM-receptor interaction	34	1.98E-03
hsa04310	Wnt signaling pathway	52	1.98E-03
hsa05215	Prostate cancer	36	1.98E-03
hsa04810	Regulation of actin cytoskeleton	72	2.25E-03
hsa04360	Axon guidance	61	2.46E-03
hsa04919	Thyroid hormone signaling pathway	44	2.73E-03
hsa04540	Gap junction	35	2.82E-03
hsa04510	Focal adhesion	67	4.17E-03
hsa00532	Glycosaminoglycan biosynthesis	12	4.84E-03
hsa05218	Melanoma	29	5.06E-03
hsa04151	PI3K-Akt signaling pathway	102	7.16E-03
hsa00600	Sphingolipid metabolism	21	7.29E-03
hsa05220	Chronic myeloid leukemia	29	7.64E-03

We next investigated the role of KDM inhibition on AR protein expression to understand whether loss or reduction of AR expression contributed to observed effect on AR regulated genes. KDM1A, which also has H3K3 demethylase activity and is known to play a role in AR regulation (Cai et al., 2011), was used as a comparison to KDM5 inhibition but to also assess the contribution of combined inhibition on AR levels. We compared the effect of the KDM1A inhibitor, namoline (Willmann et al., 2012), alone and in combination with CPI-455, on AR expression. Interestingly, inhibition of KDM5B demethylase function, did not alter AR expression in LNCaP cells (Figures 4E, F), even though *AR* mRNA was downregulated by CPI-455 treatment (Supplementary Table S2B). Loss of AR expression was observed with KDM1A inhibition in LNCaP cells. Crucially, combined pharmaco-inhibition of KDM1A and KDM5B by namoline and CPI-455 suppressed AR expression in LNCaP:C4-2 (Figures 4G, H). To assess why loss of *AR* lead to no change in AR protein levels with CPI-455 treatment in LNCaP cells, we utilized the RNA-Seq data to examine SP1 transcription factor. SP1 is known to regulate *AR* expression (Hay et al., 2015). From the RNA-Seq data *SP1* expression was itself significantly downregulated in CPI-455 treated cells (log2 fold change 1.91, FDR-p value 1.03E-22; Supplementary Table S2A), potentially contributing to reduced transcriptional activation of *AR*. These findings support KDM1A and KDM5B cooperate in both AR expression and function, and thus pharmacological inhibition of KDM1A and KDM5B impairs androgen signaling via direct effects on the AR: KDM coregulator complex activity, and expression of AR.

### 3.4 Functional inhibition of KDM5 demethylases affects alternative gene splicing

**FIGURE 5:**
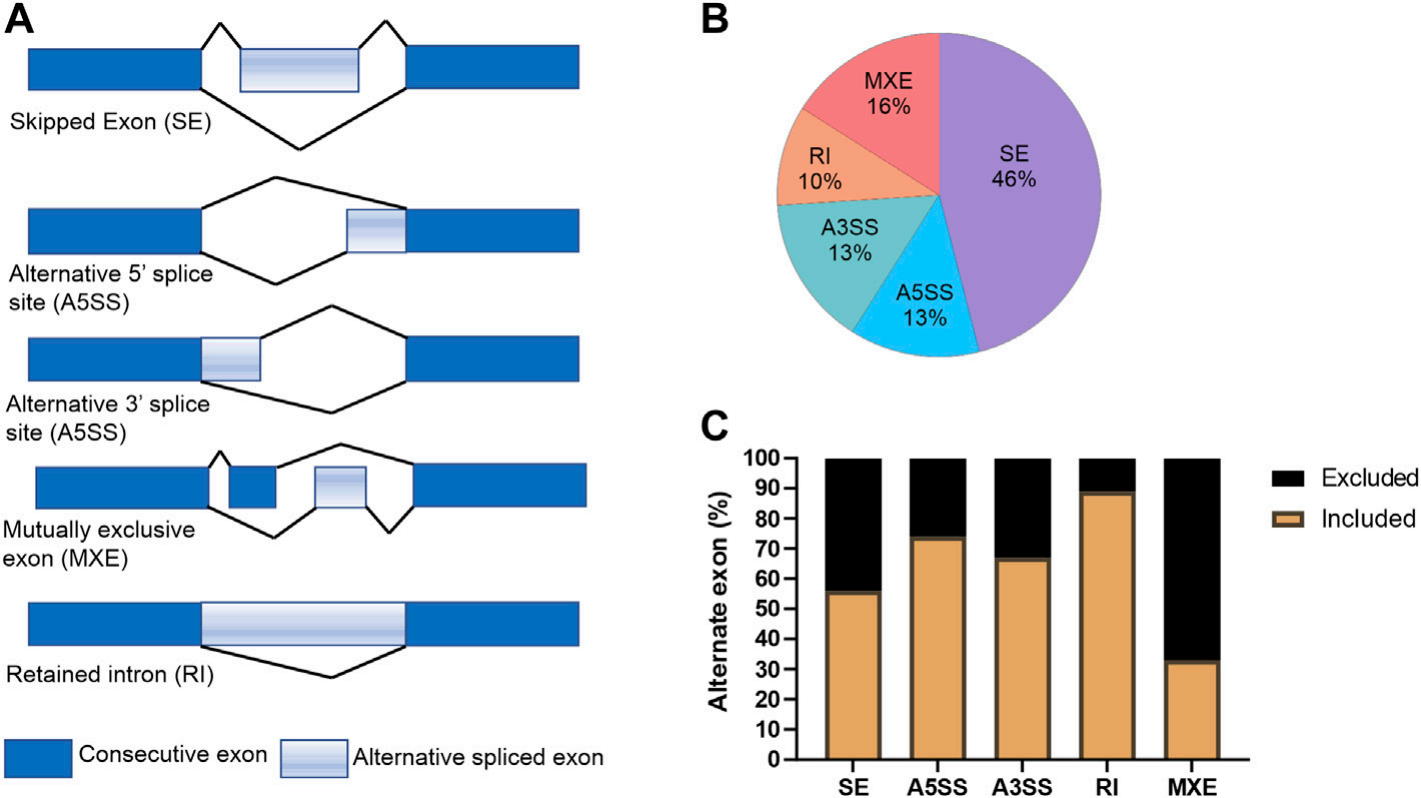
Alternative splicing events in CPI-treated LNCaP cells. **(A)** Graphical representation of the alternative splicing events. **(B)** Percentage of each splicing event for the 13088 differential spliced variants between vehicle and CPI-455 treated LNCaP cells. **(C)** Graph showing the percentage of inclusion and exclusion of the exon cassette/exon upon CPI-455 treatment for each splice event.

Recently, KDMs have been shown to play critical roles in splicing of AR (Duan et al., 2019) and KDM5B has been shown to play a role in alternative splicing in embryonic stem (ES) cells (He and Kidder, 2017). For this reason, we used the rMATs tool (Shen et al., 2014) to analyze RNA-seq data to evaluate the effect of KDM5 inhibition on alternative splicing events in CPI-455 treated LNCaP cells (Figure 5A). Upon CPI-455 treatment 13088 significant alternative splicing events were identified (absolute delta % spliced in (dPSI) ≥5%, FDR <0.05; Supplementary Table S3). The most common splicing events were skipped exons (46%, Figure 5B). Interestingly, when examining whether the alternative spliced cassette (exon/intron) were included/excluded upon CPI-455 treatment, we identified that 89% of retained introns occurred upon KDM5-inhibition (Figure 5C). A total of 5440 genes were associated with the 13088 alternative spliced events. On further investigation of the genes that had an alternative spliced event, 1880 genes (12.3% of total number of genes) were also differentially expressed. Pathway analysis of the 3560 genes that were only alternatively spliced, were involved in ribosome biogenesis, splicesome, cell cycle, and DNA repair. We also performed pathway analysis on the genes with each splicing. Variations in the pathways were identified, for example, genes with retained introns were involved in splicesome whereas genes with skipped exons were involved in cell cycle and alternative 3’ splice site (A3SS) in DNA repair (Supplementary Table S4B–F).

These findings show that CPI-455 dramatically alters splicing suggesting KDM5 play a role in alternative splicing. Further study of the effect of CPI-455 on genome-wide distribution of H3K4me3 and how this influences alternative splicing is warranted.

### 3.5 CPI-455 KDM5 inhibitor reduces cell proliferation of PCa cells and invasion in a subset of CRPC

**FIGURE 6:**
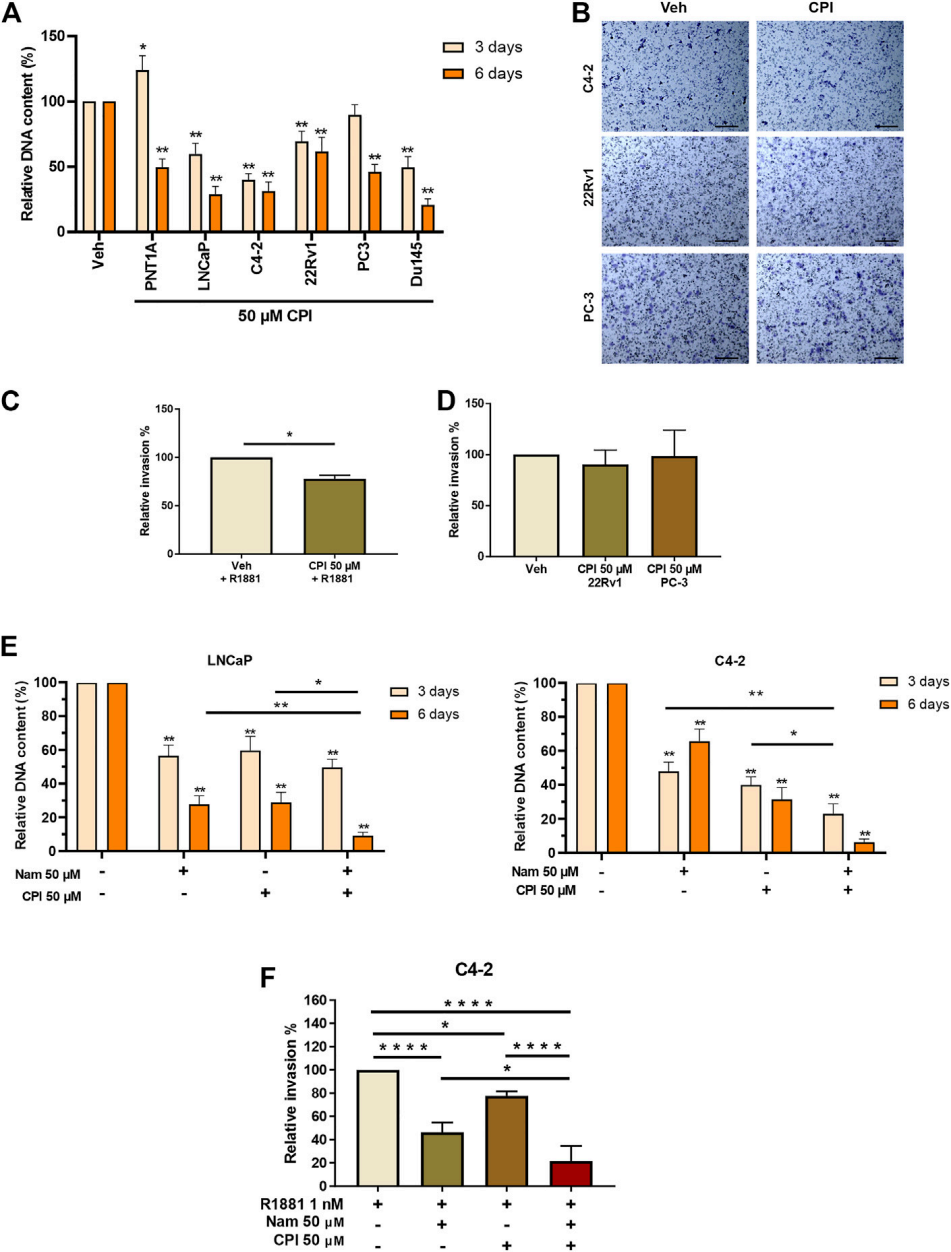
Phenotypic effects of KDM5 inhibitor and KDM1A inhibitor combination treatments in PCa cell lines. **(A)** Proliferation was quantified by examining the relative DNA content between Vehicle control (0.1% DMSO) and CPI-455 (50 µM) treatment in non-malignant PNT1A and PCa cell lines, LNCaP, LNCaP:C4-2, 22RV1, PC3, and DU145. Measurements were taken at 3 and 6 days after treatment. **(B)** Representative images of invasion assays of vehicle control and CPI treatment for PCa cell lines LNCaP:C4-2, 22RV1, and PC-3. Scale bar represents 200 µM. Cells were fixed and stained 24 h after inhibitor treatment. Relative percentage invasion to vehicle control is shown **(C)** LNCaP:C4-2 and **(D)** 22RV1 and PC-3. **(E)** Proliferation assay showing the relative DNA content 3 and 6 days after inhibitor combination treatment with CPI-455 (50 µM) and Namoline (50 µM). **(F)** Invasion assay for inhibitor drug combinations for LNCaP:C4-2. Veh–Vehicle control 0.1% DMSO, Nam -Namoline, CPI–CPI-455. Significance shown by *p*-values *p ≤ 0.05, **p ≤ 0.005, ***p ≤ 0.001, *p ≤ 0.0001.

We next examined the effect of CPI-455 on PCa cell proliferation and invasion. AR positive castrate sensitive (LNCaP), castrate resistant (LNCaP: C4-2 & 22RV1) and AR negative (PC3 & DU145) PCa cell lines, were treated with the CPI-455, for 3 days (72 h) and 6 days (144 h). Interestingly, LNCaP, LNCAP: C4-2, 22RV1 and DUI45 CPI-455 treated cells all showed loss of proliferation at 3 days compared to vehicle treated cells (Figure 6A). No loss of proliferation was seen at 3 days for PNT1A (epithelial cells) or PC3 (CRPC cells), but loss of proliferation of all cells was seen at 6 days (Figure 6A), suggesting that KDM5 demethylase function are important for cell proliferation. We next investigated the effect of KDM5B inhibition on LNCaP:C4-2, 22RV1 and PC3 using *in vitro* cell invasion assays (Figure 6B). CPI-455 reduced invasion in LNCaP: C4-2, but not in 22RV1 and PC3 (Figures 6C, D).

The individual and combined effects of KDM1A (namoline) and KDM5B (CPI-455) inhibition on LNCaP and LNCaP: C4-2 proliferation were then investigated. While both namoline and CPI-455 individually reduced LNCaP and LNCaP:C4-2 proliferation (Figure 6E), combined KDM1A and KDM5B inhibition further reduced cell proliferation. Consistent with this, combined KDM1A and KDM5 inhibition also reduced *in vitro* invasion of LNCaP:C4-2 (Figure 6F). These findings show that KDM5B inhibition causes loss of proliferation in PCa cells and invasion of AR-driven CRPC cells. Combined inhibition of KDM5B and KDM1A enhanced these effects, suggesting that KDM1A and KDM5B cooperate in promoting pro-metastatic phenotypes.

## 4 Discussion

The regulation of H3K4 di- and tri-methylation by lysine demethylases such as KDM1A and KDM5 family is a crucial mechanism for gene expression [reviewed in (Zhao and Shilatifard, 2019)]. Both KDM1A and KDM5B can interact directly with AR (Metzger et al., 2005; Xiang et al., 2007), both function as AR coregulators that promote androgen signaling, and both regulate AR expression (Xiang et al., 2007; Cai et al., 2011; Casati et al., 2013; Cai et al., 2014). The importance of KDM1A in PCa is well established (Metzger et al., 2005; Kahl et al., 2006; Wissmann et al., 2007; Cai et al., 2011; Kashyap et al., 2013; Nilsson et al., 2015). The functional significance of KDM5B in PCa cells, and the therapeutic feasibility and clinical utility of KDM5 inhibitors to treat PCa remain poorly understood. There is also increasing evidence that KDM5B also plays complex and context specific roles in prostate development, homeostasis and carcinogenesis (Xiang et al., 2007; Klein et al., 2014; Li et al., 2020; Liu et al., 2020; Yang et al., 2020).

Recent studies utilizing a prostate specific KDM5B conditional knockout mouse model indicates KDM5B loss contributes to prostate hyperplasia (Liu et al., 2020). However, *KDM5B* mRNA expression is higher in PCa patient specimens (Liu et al., 2020) and KDM5B promotes proliferation and invasion and is associated with worse clinical parameters of poorer outcomes (Yang et al., 2020). Consistent with a pro-oncogenic function for KDM5B, prostate-specific depletion of KDM5B and PTEN in the mouse prostate epithelium and PCa cell lines supports a role for KDM5B in activating the PI3K/AKT pathway in prostate carcinogenesis (Li et al., 2020). Collectively these results suggests that KDM5B functions to limit prostate hyperplasia during prostate development but can promote prostate carcinogenesis via PI3K/AKT and androgen signaling. These studies highlight the complexity and context specific functions of KDM5B in the prostate.

Here we sought to extend the understanding of the individual and cooperative roles and clinical relevance of KDM1A and KDM5B in prostate adenocarcinoma and neuroendocrine tumor specimens. While KDM5B expression is frequently altered in PCa patients, only five PCa patients were found to harbor KDM5B mutations. Approximately ∼5–6% of PCa patients exhibited *KDM5B* copy number amplification, whereas <1% of patients possessed heterozygous KDM5B deletion. This suggest that the increased KDM5B mRNA and protein expression observed in PCa is attributable to transcriptional upregulation as opposed to copy number amplification. Collectively this data suggests KDM5B is necessary for PCa cell viability and contributes to prostate carcinogenesis.

Our study quantified cytoplasmic and nuclear KDM5B expression in non-malignant prostate, prostate adenocarcinoma and neuroendocrine tumor specimens. Nuclear expression of KDM5B was positively correlated with AR expression in both non-malignant and PCa. These findings correspond to our *in vitro* findings indicating important roles for KDM5B as an AR coregulator in androgen dependent PCa. KDM5B was detectable in metastatic adenocarcinoma and neuroendocrine Tumors, only a minority of this cohort (40% of cases) and further investigation is required into the role in these advanced PCa.

Although little is known about the cytoplasmic functions of KDM5B, its presence in the cytoplasm has been linked to the cell cycle and CDK1 function, where CDK1-mediated KDM5B phosphorylation localized KDM5B to the nucleus. CDK1 activity is highest at G2/M phase and following CDK1 knockdown, a proportion of KDM5B nuclear protein re-localised to the cytoplasm (Yeh et al., 2019). Elevated KDM5B cytoplasmic expression was noted in metastatic tumors (Figure 2K). SKP2 was previously shown to be elevated in PCa progression which in-turn regulated TRAF E3 ubiquitin ligase ubiquitylation of KDM5B which led to loss of demethylase function, nuclear re-localisation of KDM5B and increase in H3K4me3 (Lu et al., 2015). Therefore, it is possible that cytoplasmic KDM5B localization is related to protein degradation (Hendriks et al., 2015).

Consistent with KDM5B protein expression in the PCa tumor specimens reported here, *KDM5B* mRNA expression was higher in both androgen dependent (LNCaP) and androgen-independent (LNCaP: C4-2, 22Rv1, PC3) cell lines as compared to the non-malignant PNT1A prostate cell line. KDM5B mRNA and protein expression was highest in androgen dependent LNCaP (Figure 1E). Given the recently identified role for KDM5B in PI3K/AKT activation, it is possible that castrate resistant PCa cells with high constitutive PI3K/AKT no longer require elevated KDM5B.

Whether the use of KDM5B selective inhibitor, CPI-455 was a viable drug therapy option for PCa (Vinogradova et al., 2016).

Our study also assessed whether the use of CPI-455 KDM5 inhibitor may provide preclinical mechanistic evidence supporting future development of KDM5B inhibitors to suppress androgen signaling in PCa (Vinogradova et al., 2016). CPI-455 induced dramatic reprograming of the androgen regulated transcriptome in LNCaP including genes associated with cancer pathways, supporting a tumor promoting role for KDM5B in PCa (Supplemental file 1A). The CPI-455 reduced expression of AR regulated genes and AR mRNA (Supplementary Table S2B). While AR mRNA expression was reduced, AR protein expression persisted in LNCaP. Thus, the effect of CPI-455 on androgen signaling is likely attributable to inhibition of the AR-coregulator function of KDM5B, but a potential indirect role of KDM5A or KDM5C cannot be excluded and further investigated.

CPI-455 also induced dramatic reprogramming of alternative splicing in LNCaP affecting genes associated with splicing and DNA repair pathways (Supplemental File S3A). The importance of KDM5B in the maintenance of genome stability and in DNA damage response, recruiting Ku70 and BRCA1 to sites of damage is established (Li et al., 2014). Whether the alternative splicing of DNA repair genes was due to direct or indirect regulation by KDM5B, or due to an increase in genome instability due to increased genome wide levels of H3K4me3 caused by inhibition of KDM5 family, is not clear. But KDM5B is known to play important roles in genome stability and KDM5B inhibition and concomitant increases in H3K4me3 has been shown to increase sensitivity to radiation therapy by impairing DNA repair response (Bayo et al., 2018). KDM5B has been previously shown to be involved in alternative splicing in mouse embryonic cells (He and Kidder, 2017). As with our study, this study showed KDM5B depletion resulted in altered expression of splice variants (He and Kidder, 2017).

The CPI-455 inhibitor significantly reduced proliferation of malignant prostate cells (LNCaP, LNCaP:C4-2, 22Rv1, and Du145), where non-malignant PNT1A prostate epithelial cells were less sensitive to CPI-455at 3 days, suggesting the KDM5 family is essential for PCa cell proliferation (Figure 6A). This is consistent with the context specific function of KDM5B identified in the KDM5B prostate specific knockout mouse (Liu et al., 2020) and with the reported cell cycle functions of KDM5B (Yamane et al., 2007; Hayami et al., 2010). Studies have also shown knock-down KDM5A and KDM5C leading to loss of proliferation (Hong et al., 2019; Du et al., 2020). CPI-455 had no significant effect on invasion of LNCaP: C42 or 22Rv1. We also examined the effect of CPI-455, alone and in combination with the KDM1A inhibitor, namoline, on PCa proliferation and invasion. While namoline alone inhibited both PCa cell invasion and proliferation, the combined inhibition of KDM1A and KDM5B significantly increased the inhibition of proliferation and invasion of LNCaP: C42 cells, over the effect achieved by either inhibitor on its own (Figure 6).

With recent multicenter studies utilizing epigenomics to stratify advanced PCa, understanding chromatin remodeling factors, how their expression is altered and how this changes the epigenomic landscape are crucial for identifying new therapeutic targets (Armenia et al., 2018; Abida et al., 2019; Zhao et al., 2020; Tang et al., 2022). This study identifies KDM5B as histone modifier that is crucial for AR regulated gene expression in castrate sensitive PCa, but whose role in advanced cancers is altered. Our study shows the potential benefit of KDM5B selective inhibitor in treatment of AR-positive PCa. Additionally, this study has identified cooperative functional interaction of KDM1A and KDM5B in regulation of AR expression and PCa phenotype. Further studies are now warranted to establish the individual and cooperative genome-wide epigenetic roles of KDM1A and KDM5B. Given the importance of epigenetic context in determining the coactivator and corepressor functions of KDM1A (Metzger et al., 2010), this will inform whether the epigenetic context of these loci governs which KDM regulates H3K4 methylation at specific loci. Such insights will mechanistically underpin future studies into whether combination therapies targeting KDM1A and KDM5B may be beneficial in CRPC patients.

## Data Availability

The datasets presented in this study can be found in online repositories. The names of the repository/repositories and accession number(s) can be found below: GEO accession number: GSE194278.
