# Supplementary material for: The KDM5B and KDM1A lysine demethylases cooperate in regulating androgen receptor expression and signalling in prostate cancer
Source: Front Cell Dev Biol. 2023 Apr 19;11:1116424. doi: 10.3389/fcell.2023.1116424 (PMC10154691; doi:10.3389/fcell.2023.1116424)
Supplement: Supplementary file 5 [file DataSheet1.docx]

**Supplementary Figure 1.**

A) KDM5B mRNA varies greatly in patients with diploid copy number and is higher in patients with copy number gain (GISTIC). B) KDM5B is not androgen regulated in PCa cell lines. QRT-PCR of *KDM5B* levels (normalised to *GAPDH)*. Androgen induction did not change the expression of *KDM5B* in LNCaP and C4-2.


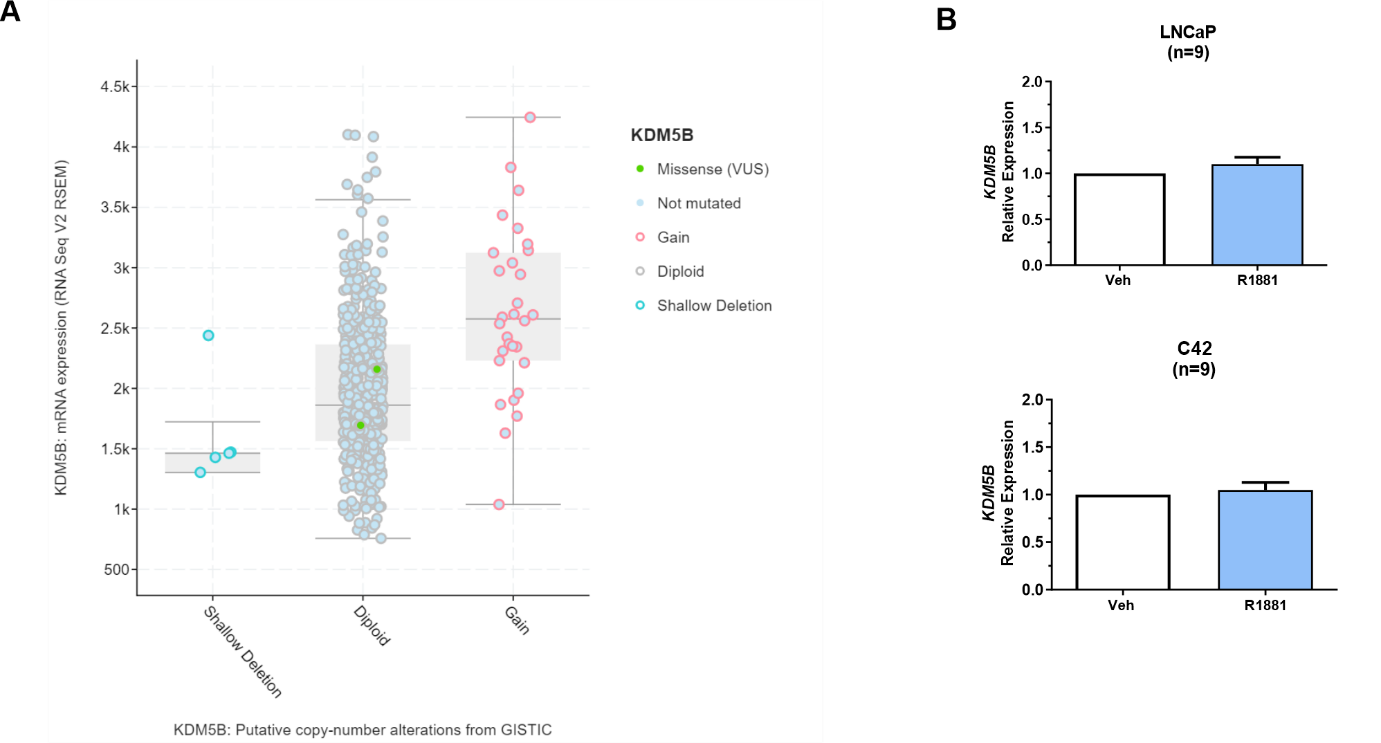


**Supplementary Figure 2.**

KDM5B protein immunohistochemistry was used in non-malignant (n = 43) and prostate cancer (n = 97) specimens. Representative KDM5B staining of normal tissue (A), tumour tissue (B) and less differentiated tumour tissue (C; Scale bars: 50 μM). (D) H-score evaluation of KDM5B cytoplasmic staining cytoplasmic H-score, low = 25-50, medium = 55-75, high = 80-150. Statistical p-values were determined by χ2-test KDM5B H-scores were correlated with biochemical recurrence using Kaplan Meier estimate (n = 30) for both cytoplasmic (E) and Nuclear (F) staining. Nuclear H-score: low = 0-20, medium = 25-40, high = 45-155; Cytoplasmic H-score: low = 25-50, medium = 55-75, high = 80-150; BCR = Biochemical recurrence. Statistical analysis was performed with log-rank test.

**
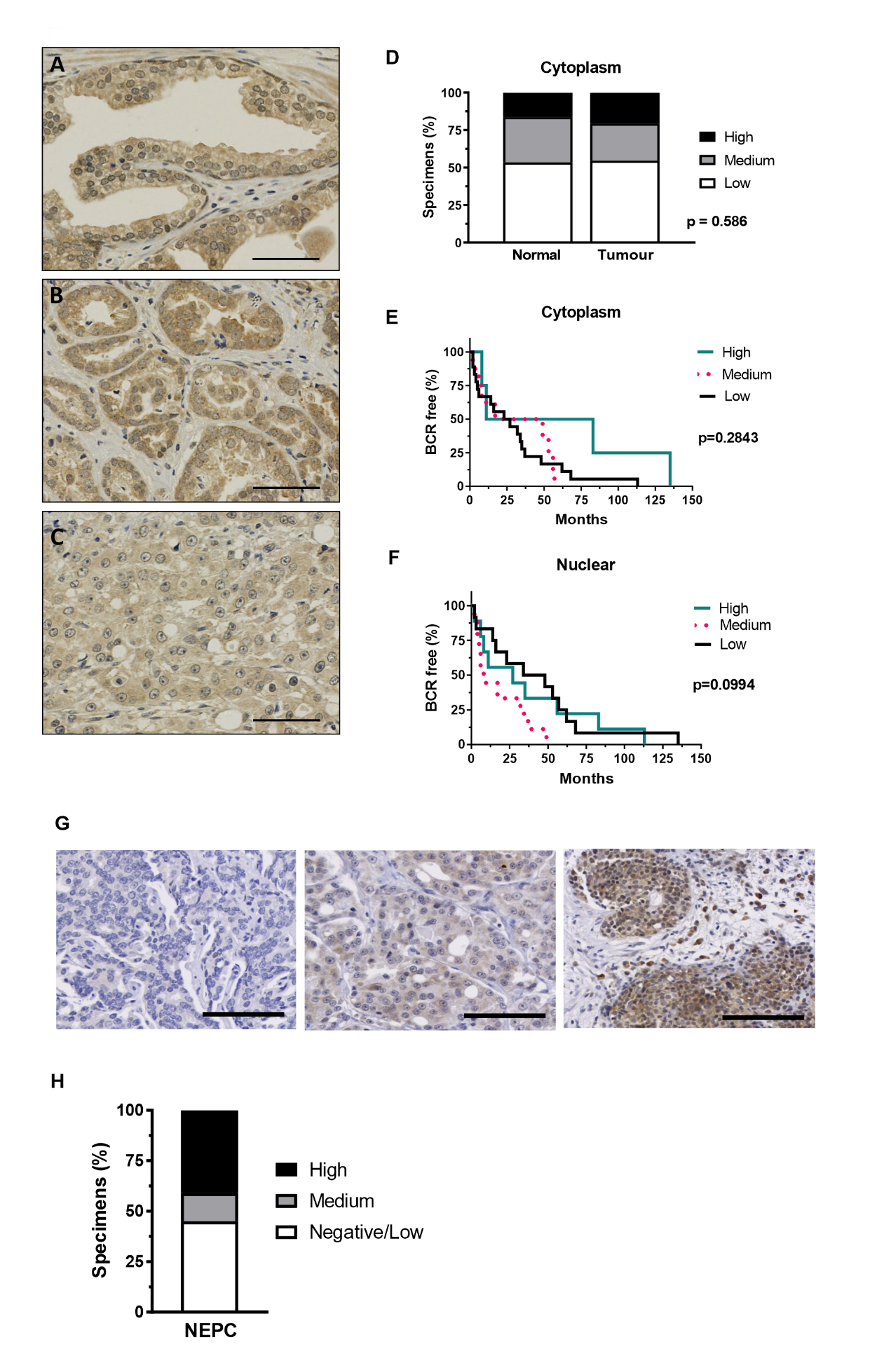
**

**Supplementary Table 1**

**PRAD-TCGA differential gene analysis between tumours with low and high levels of *KDM5B***

(A) Differentially expressed genes lower in Q1 (higher in Q4) and higher in Q1 (lower in Q4). Genes shown are significantly different between the two groups (Q1, low *KDM5B* and Q4, high *KDM5B*) log2 fold change ≥ 1 and FDR p value < 0.05. (B) Pathways identified from the differential expressed genes.

**Supplementary Table 2**

**Differential gene expression analysis between vehicle and CPI-455 treated LNCaP cells**

(A) Significant differential genes between vehicle and CPI-455 treated LNCaP cells had log2 fold change ≥ 1 and FDR p value < 0.05. (B) *AR* and AR target gene expression changes between vehicle and CPI-455 treated LNCaP cells.

**Supplementary Table 3**

Differentially expressed splicing variants between vehicle and CPI-455 for each splicing event

**Supplementary Table 4**

Pathway analysis of the alternative splice events between vehicle and CPI-455 treated LNCaP cells.
